# Prognostic influences and prediction model construction for traumatic cervical spinal cord injury

**DOI:** 10.3389/fneur.2025.1684409

**Published:** 2026-01-09

**Authors:** Xin Kang, Xiao-bo Zhang, Xi-dan Gao, Zi-lin Gao, Zheng-wei Xu, Yingang Zhang

**Affiliations:** 1 Department of Orthopaedics, First Affiliated Hospital, Medical College of Xi’an Jiaotong University, Xi'an, Shaanxi, China; 2 Department of Spine Surgery, Honghui Hospital, Xi’an Jiaotong University, Xi'an, Shaanxi, China

**Keywords:** traumatic cervical spinal cord injury, prognosis, predictive model, efficacy assessment, retrospective analysis

## Abstract

**Study design:**

Retrospective analysis of prognostic factors in traumatic cervical spinal cord injury (TCSCI) patients.

**Objective:**

Explore a novel approach for the prognosis assessment of TCSCI.

**Methods:**

A retrospective analysis of clinical data was conducted on patients who underwent operation at Xi’an Honghui Hospital between March 2016 and May 2023. The study included sex, age, hypertension, diabetes mellitus (DM), damage range, ossification of the posterior longitudinal ligament (OPLL), cervical spinal canal stenosis grading (CSCS), surgical timing, and postoperative complications (deep vein thrombosis, pulmonary complications, pressure ulcers, and urinary tract infections). A prognostic model was established by these differential factors and evaluating the sensitivity and specificity for predicting paralysis occurrence.

**Results:**

Paralysis was found to be 4.009 times more likely in patients with DM compared to those without (95% CI: 1.250–12.863, *p =* 0.020). Patients with diffuse damage had a 7.912 times higher likelihood of developing paralysis than those without diffuse damage (95% CI: 3.466–18.063, *p* < 0.001). Patients with CSCS grade III had an 8.862 times higher likelihood of developing paralysis compared to those with grades 0, I, or II (95% CI: 3.280–23.946, *p* < 0.001). The probability of paralysis with ≥2 postoperative complications was 4.625 times greater (95% CI, 1.107–19.323, *p =* 0.036) than without complications. The probability of paralysis in patients with surgical times >3 days was 3.132 times greater (95% CI, 1.325–7.407, *p =* 0.036) than within 3 days (95% CI, 1.325–7.407, *p =* 0.009). The CSCS had the greatest effect on the prognosis of patients with paralysis, followed by the damage range. Sex, age, hypertension, and OPLL were not associated with prognosis (*p* > 0.05).

**Conclusion:**

The prognosis of TCSCI is influenced by factors including DM, damage range, CSCS, surgical timing, and the number of postoperative complications. A clinical prognostic model was developed based on these prognostic factors, demonstrating a strong predictive capability for paralysis.

## Introduction

Recently, traumatic cervical spinal cord injury (TCSCI) has emerged as a global health risk due to its high incidence and mortality rates (1). The incidence of TCSCI varies with the level of economic development across different countries and periods, ranging from 12.1 to 57.8 cases per million in high-income countries and from 12.7 to 29.7 cases per million in low-income countries. The medical costs associated with TCSCI are substantial, necessitating complex medical support and thereby exacerbating the economic burden on individuals and society.

Although various assessment tools exist for evaluating patients’ neurological function, such as the International Standards for Neurologic Classification of Spinal Cord Injury (ISNCSCI) and the American Spinal Injury Association (ASIA) Impairment Scale (AIS), there is still no consensus on the optimal approach for measuring these outcomes. The ISNCSCI offers consistent SCI classification terminology and definitions for detecting changes in neurological function over time (2) and is applicable in acute care, recovery, and outpatient settings (3). In contrast, the AIS classification is subject to certain limitations. These include the possibility of misclassification at initial examination or follow-up, which may result in a change from a neurologically intact state to an incomplete state, and vice versa, based only on sacral sparing changes (4–6). They only examined the possibility of paralysis in patients at a single level, either preoperatively or postoperatively. Additionally, comprehensive summaries of risk indicators affecting patient prognosis are lacking, as different TCSCI patients may have different prognoses based on sex, injury mechanism, and degree of injury (7–9).

This study summarized patients’ admission information, injury conditions, preoperative imaging data, and postoperative complications. Independent risk factors were identified through statistical methods, with all procedures meticulously adhering to experimental principles to minimize uncertainty and subjectivity. This approach aims to provide more comprehensive and effective treatment plans and recovery strategies for TCSCI patients.

## Methods

### Object

This study was approved by the ethics committee of Honghui Hospital (202405009). The clinical data of 264 TCSCI patients from March 2016 to May 2023 at Honghui Hospital, Xi’an, were retrospectively analyzed. The inclusion criteria were age ≥ 12 years, cervical SCI with no missing clinical or imaging data, traumatic cause of injury, and no obvious contraindication to surgery. The exclusion criteria were concomitant systemic diseases, such as thoracolumbar SCI, cavernous spinal disease, traumatic brain injury, cognitive impairment, loss to follow-up or death, contraindications to surgery, pregnancy or lactation.

**Figure 1:**
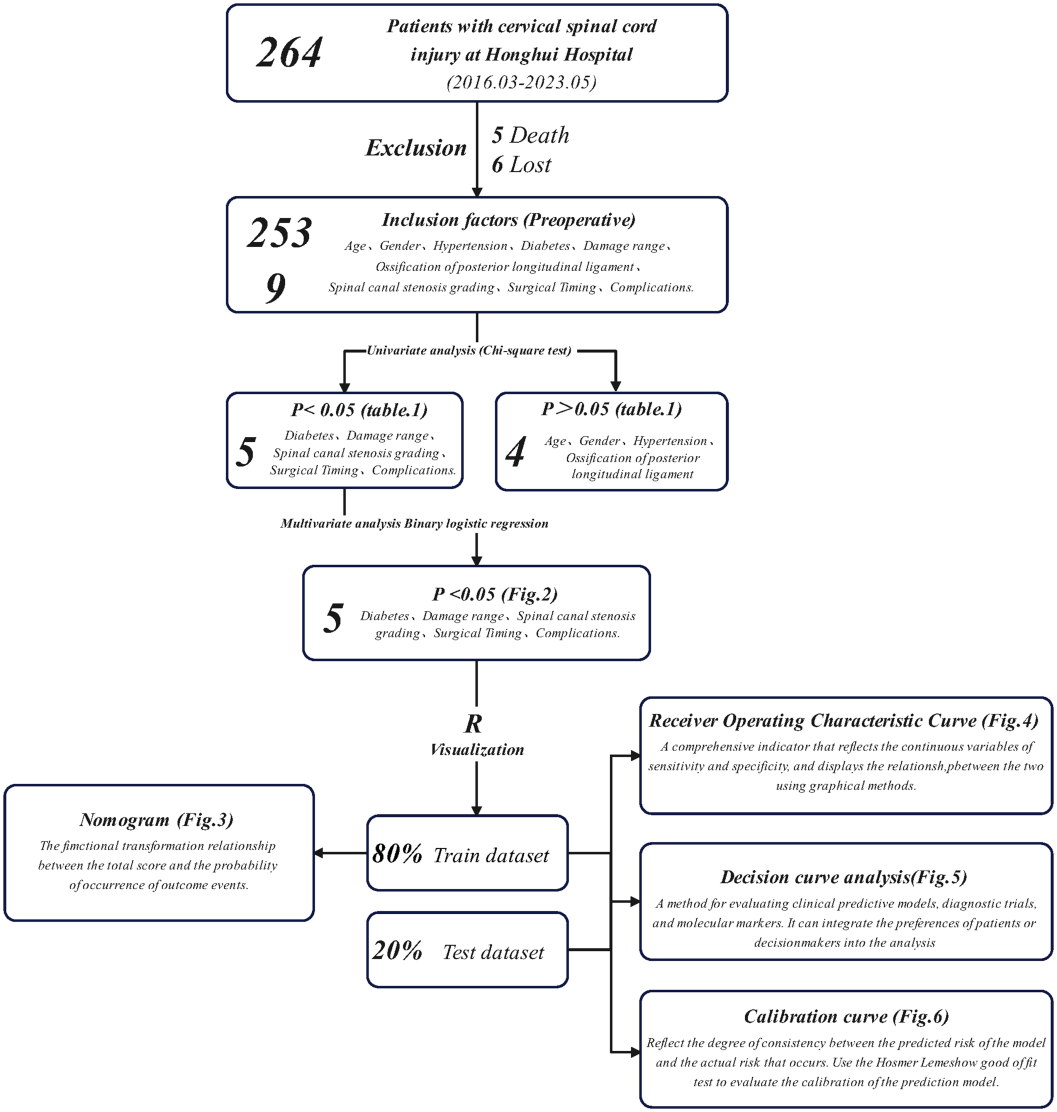
The workflow of the TCSCI clinical prognostic model.

Five patients died within 1 year of discharge, and 6 had missing follow-up data, resulting in the inclusion of 253 TCSCI patients. They were randomly assigned to a training set (203, 80%) or a validation set (50, 20%) (Figure 1). The training set data were used to construct a visual column-line graphical model. The accuracy of the prognostic model for the occurrence of paralysis was estimated by the area under the curve (AUC) of the subjects’ operating characteristic (ROC) curve and bootstrap resampling method (500 iterations), with 0.5–0.7 representing poor discriminatory ability of the model, 0.7–0.9 representing better discriminatory ability of the model, and >0.9 suggesting that the model discriminates very well. In addition, the Hosmer–Lemeshow (H-L) test was used to construct calibration plots to evaluate the consistency of the probabilities and real situations. Finally, decision curve analysis (DCA) was used to evaluate the net benefit of the clinical prognostic model under tolerable risk and clinically applicable fit, i.e., whether the model was worth being applied.

### Indicators

A comprehensive overview of TCSCI patients was compiled, encompassing demographic data such as sex, age, comorbidities (e.g., hypertension and diabetes mellitus), and extent of injury. Additionally, the degree of ossification of the posterior longitudinal ligament (OPLL) and the severity of spinal stenosis were recorded from imaging findings. The MRI classification of spinal canal stenosis grading (CSCS) followed Muhle et al.’s (10) three-level quadratic approach. Due to its lack of consideration for alterations in spinal cord signals, this study employed the new grading criteria proposed by Kang et al. (11) in 2011. The criteria classify conditions into four grades based on T2-weighted sagittal images: grade 0 indicates no significant spinal stenosis or subarachnoid compression <50%; grade 1 indicates >50% subarachnoid compression without clear spinal cord compression; grade 2 indicates spinal cord compression and displacement without abnormal spinal cord signals; grade 3 indicates intraspinal signal with or without abnormal spinal cord signals. Grade 3 indicated an abnormal signal in the spinal cord, despite the spinal cord not being abnormal. This grading not only indicates the degree of spinal canal compression but also considers spinal cord signal and morphology. Damage range was defined by observations on MRI T2-weighted imaging: ≤1 spinal segment indicates “limited,” whereas >1 segment indicates “diffuse.” Surgical timing and postoperative complications (including deep vein thrombosis, pulmonary complications, pressure ulcers, and urinary tract infection) were documented. Since the AISA scale, akin to our prognostic model, is used for outcome prediction, its sensitivity and specificity were validated to assess its relevance. However, it was not utilized as a criterion for evaluating paralysis outcomes. Patient prognostic outcomes were assessed using the Frankel scale (12), where grades A-C were categorized as the paralysis group and grades D-E as the non-paralysis group.

### Statistical analysis

The data were analyzed in this study by IBM SPSS Statistics 26. Count data are expressed as frequencies and percentages and were analyzed using the chi-square test. Measurement data were tested for normality and chi-square tests, and those that did not conform to a normal distribution are expressed as the median and quartile M (P25, P75). Differences between groups were compared using nonparametric tests (Mann–Whitney U test). *p <* 0.05 was considered to indicate statistical significance. Variables with *p <* 0.05 were included in a binary logistic regression analysis model using stepwise regression analysis to ensure that only significant variables were included in the regression equation before each new variable was introduced. A multifactorial model (Hosmer–Lemeshow test value > 0.05) was built for variables that excluded confounding factors, and differences were considered statistically significant at *p <* 0.05 (corrected OR). Forest plots were constructed to visualize the integration of the above variables. The clinical prognostic modeling process was implemented through the R program (version 4.1.2).

## Results

Figure 1 shows the flow chart. The categorical statistics and results of the significance analysis of the patients’ conditions are shown in Table 1. Among them, there were 209 normal patients (82.6, 80.8% male) and 44 paralyzed patients (17.4, 79.5% male). As shown in the table, age (*p =* 0.514), sex (*p =* 0.841), hypertension (*p =* 0.791), and OPLL (*p =* 0.777) had less effect on paralysis; DM (*p =* 0.014), damage range (*p =* 0.025), cervical spinal canal stenosis grade (*p <* 0.001), complications (*p <* 0.001), and surgical timing (*p =* 0.004) were associated with the paralysis rate.

**Table 1:** Statistical analysis of patient data.

Prognostic indicators	Subgroup	Normal	Paralytic	X^2^	*p* value
Age	<65 years	172 (82.2%)	38 (86.3%)	0.426	0.514
	≥65 years	37 (17.8%)	6 (13.7%)		
Sex	Male	169 (80.8%)	35 (79.5%)	0.040	0.841
	Female	40 (19.1%)	9 (20.5%)		
Hypertension	No	179 (85.6%)	37 (84.0%)	0.070	0.791
	Yes	30 (14.4%)	7 (16.0%)		
Diabetes	No	195 (93.3%)	36 (81.8%)	6.037	**0.014**
	Yes	14 (6.6%)	8 (18.2%)		
Damage range	Limit	179 (85.6%)	16 (36.3%)	5.036	**0.025**
	Diffuse	30 (14.4%)	28 (68.4%)		
Ossification of posterior longitudinal ligament	No	147 (70.3%)	30 (68.1%)	0.080	0.777
	Yes	62 (29.7%)	14 (31.9%)		
Spinal canal stenosis grading	0, I, and II	195 (93.3%)	25 (56.8%)	42.655	**<0.001**
	III	14 (6.7%)	19 (43.2%)		
Postoperative Complications	No	174 (83.3%)	27 (61.3%)	17.805	**<0.001**
	1	30 (14.3%)	10 (22.7%)		
	≥2	5 (2.4%)	7 (16.0%)		
Surgical timing	≤3 days	159 (76.1%)	24 (54.5%)	8.420	**0.004**
	>3 days	50 (23.9%)	20 (45.5%)		

*p* < 0.05 indicates a statistically significant difference.

**Figure 2:**
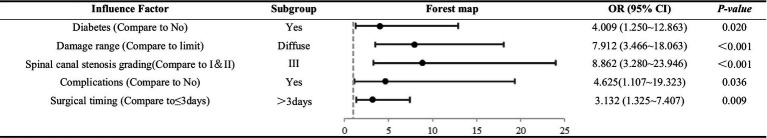
Stepwise regression analysis was used to identify five predictors and their subgroup forest plots that could independently influence paralysis.

To eliminate multicollinearity between significant individual factors, the set of predictor variables obtained in this study was optimized by stepwise regression analysis (Figure 2). As shown in the figure, five indicators independently influenced the clinical prognosis of patients who developed paralysis: DM patients were 4.009 times more likely to develop postoperative paralysis than normal patients were (95% CI: 1.250 to 12.863, *p =* 0.020). Patients with extensive injury were 7.912 times more likely to develop paralysis than were those with limited injury (95% CI: 3.466 to 18.063, *p <* 0.001), and patients with grade III spinal stenosis were 8.862 times more likely to develop paralysis than were those with grades 0, I and II (95% CI: 3.280 to 23.946, *p <* 0.001). The probability of paralysis in patients with ≥2 postoperative complications was 4.625 times greater (95% CI: 1.107 ~ 19.323, *p =* 0.036) than that in patients without complications (95% CI: 1.107 to 19.323, *p =* 0.036), and those who underwent surgery >3 days were 3.132 times more likely to be paralyzed than those who underwent surgery within 3 days (95% CI: 1.325 to 7.407, *p =* 0.009).

**Figure 3:**
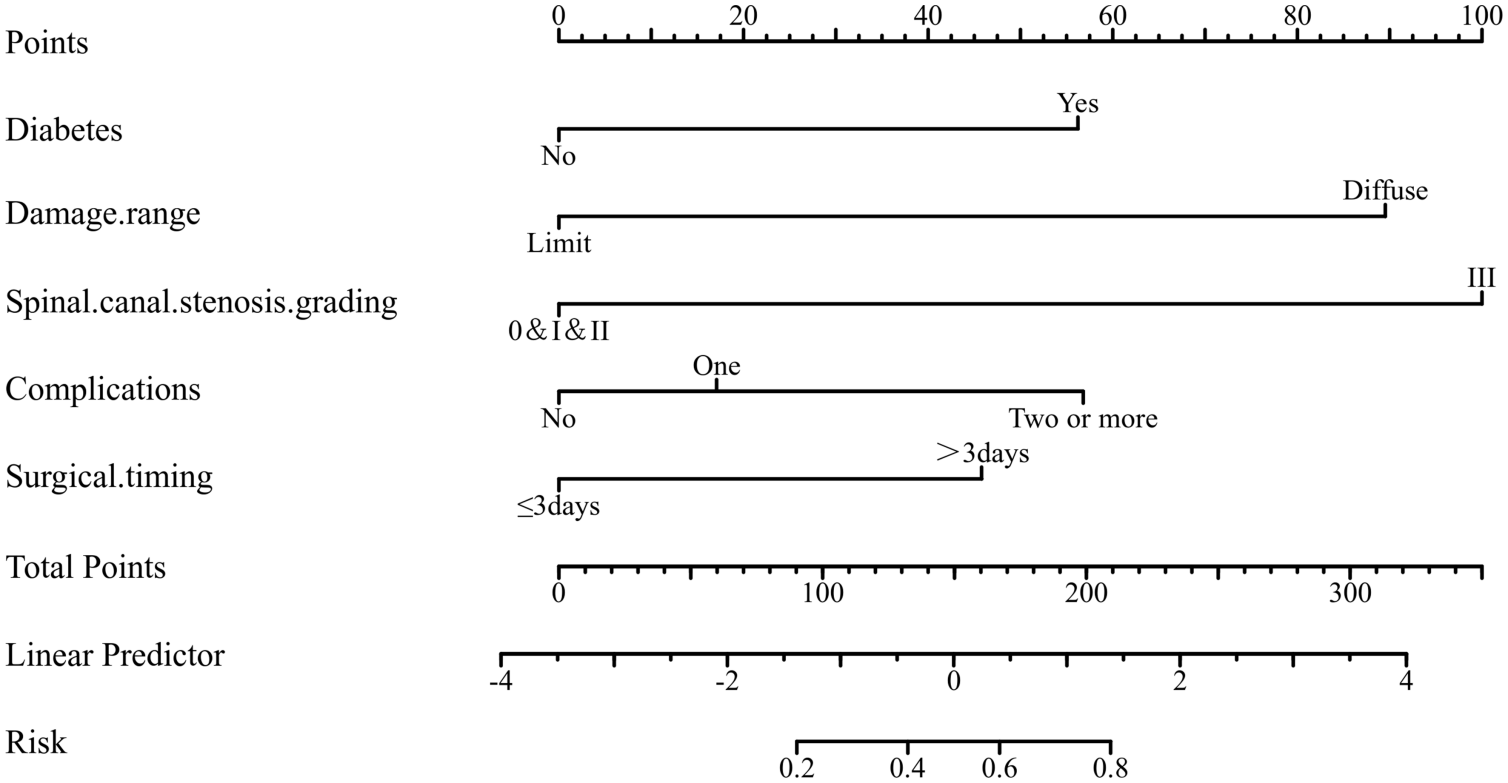
Nomogram for predicting the rate of paralysis in patients. The different states of each predictor in the validation set correspond to the points on the horizontal axis in the column line graph, the vertical line over the points can obtain the corresponding scores on the point axis, and the total scores of the five indicators correspond to the values on the horizontal axis below, that is, the probability of paralysis of the patient.

To further quantify the impact of the above metrics on the prognosis of paralysis, in this study, the hazard ratios of five independent metrics from the training set data were plotted on a column-line graph to guide the probability of postoperative paralysis in patients admitted to the hospital (Figure 3). The results showed that cervical spinal canal stenosis (CSCS) grade III had the greatest impact on patient prognosis, followed by the damage range. We can individually characterize and quantify the occurrence and prognosis of paralysis. For example, the probability of paralysis in a patient with DM admitted for limited damage, CSCS grade III, surgery within 3 days, and postoperative thrombosis is approximately 55%.

**Figure 4:**
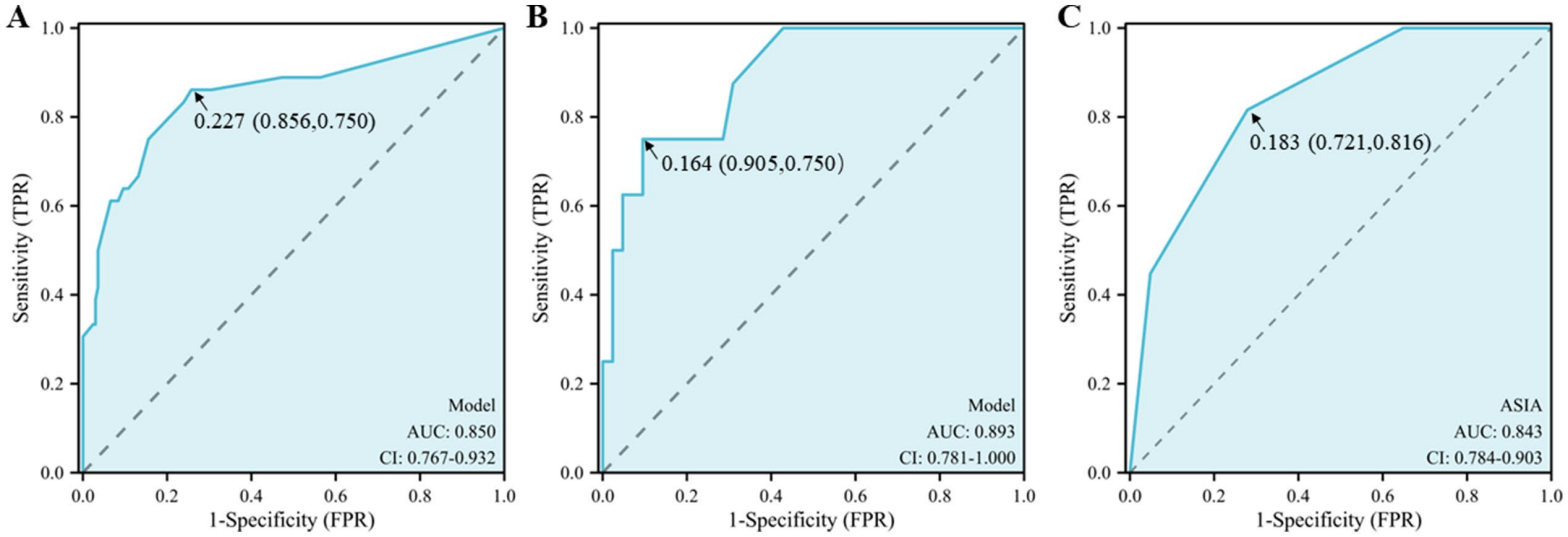
The ROC curve reflects the sensitivity and specificity. **(A)** Training set **(B)** Validation set and **(C)** ASIA dataset. The horizontal axis of the ROC curve is the false-positive rate, the vertical axis is the true-positive rate, and the closer the ROC curve is to the upper left corner, the more accurate the model prediction is.

**Figure 5:**
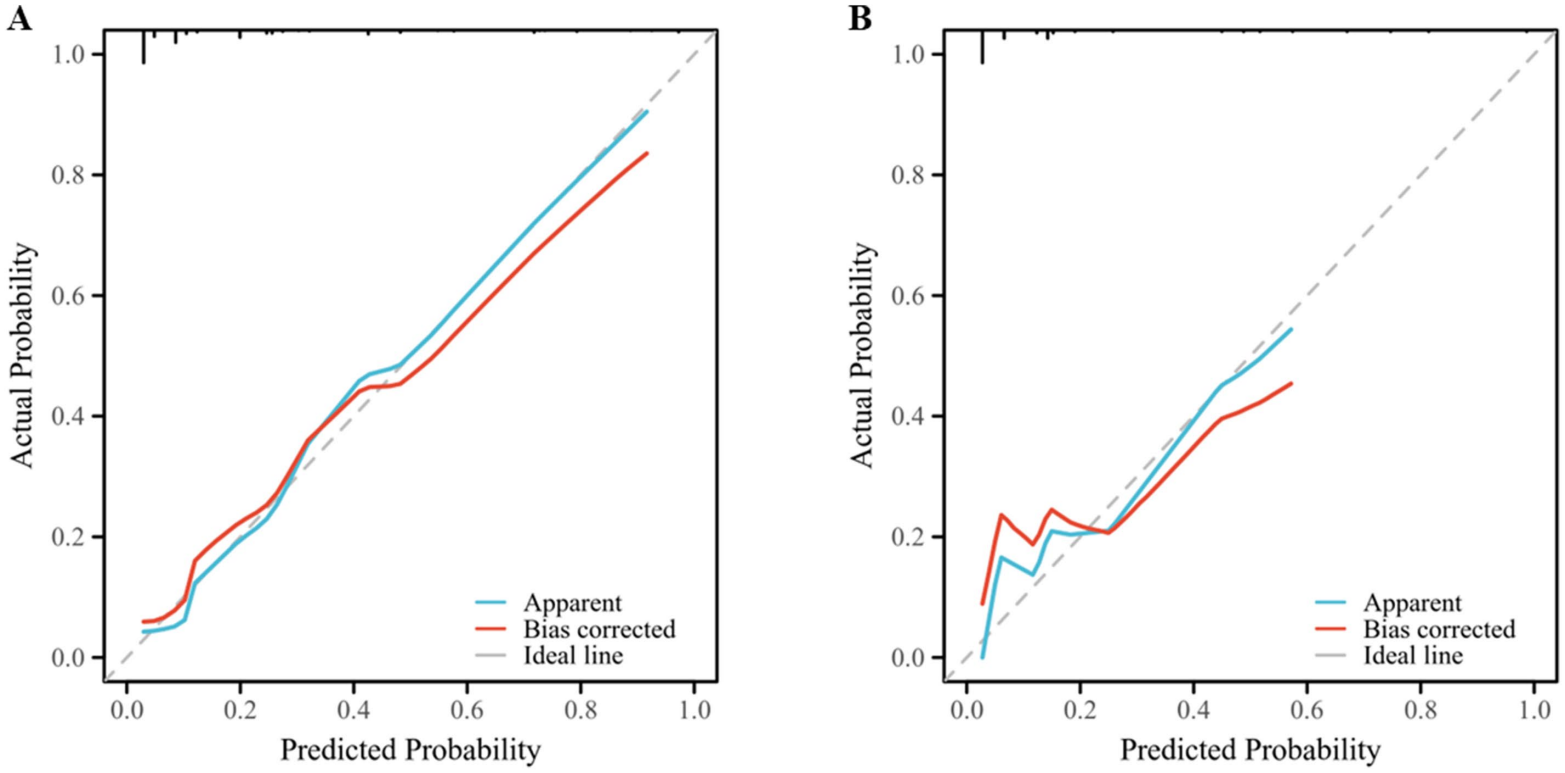
Calibration curves for the clinical prediction models. **(A)** Training set **(B)** Validation set. The X-axis is the modelpredicted probability of paralysis and the Y-axis is the actual value. The calibration curve is bias-corrected, and the diagonal ideal is the ideal curve. The greater the similarity between the corrected and ideal curves is, the better the predictive ability.

The ROC curve was used to assess the discriminative ability of the clinical prognostic model. The area under the ROC curve (AUC) of the clinical model drawn using the training set was 0.850 (Figure 4A), indicating that the model has a strong discriminatory ability. Moreover, the optimal cutoff value (i.e., cutoff value) of the model was 0.227, at which time the model had the best discriminatory ability, with a specificity and sensitivity of 0.856 and 0.750, respectively. In addition, the validation set data were used to assess the generalizability of the model. The AUC of the validation set was 0.893 (Figure 4B), the cutoff was 0.164, and the specificity and sensitivity were 0.905 and 0.750, respectively. Both of these values were greater than the specificity and sensitivity of the ASIA predictive model (0.721 and 0.816, respectively) (Figure 4C). The calibration curve, as a scatterplot visualizing the actual and predicted incidence, efficiently responded to the predictive accuracy of the column-line plot (Figures 5A,B). The training set and validation set deviated slightly from the ideal curve but still maintained a more desirable accuracy (Figure 5).

**Figure 6:**
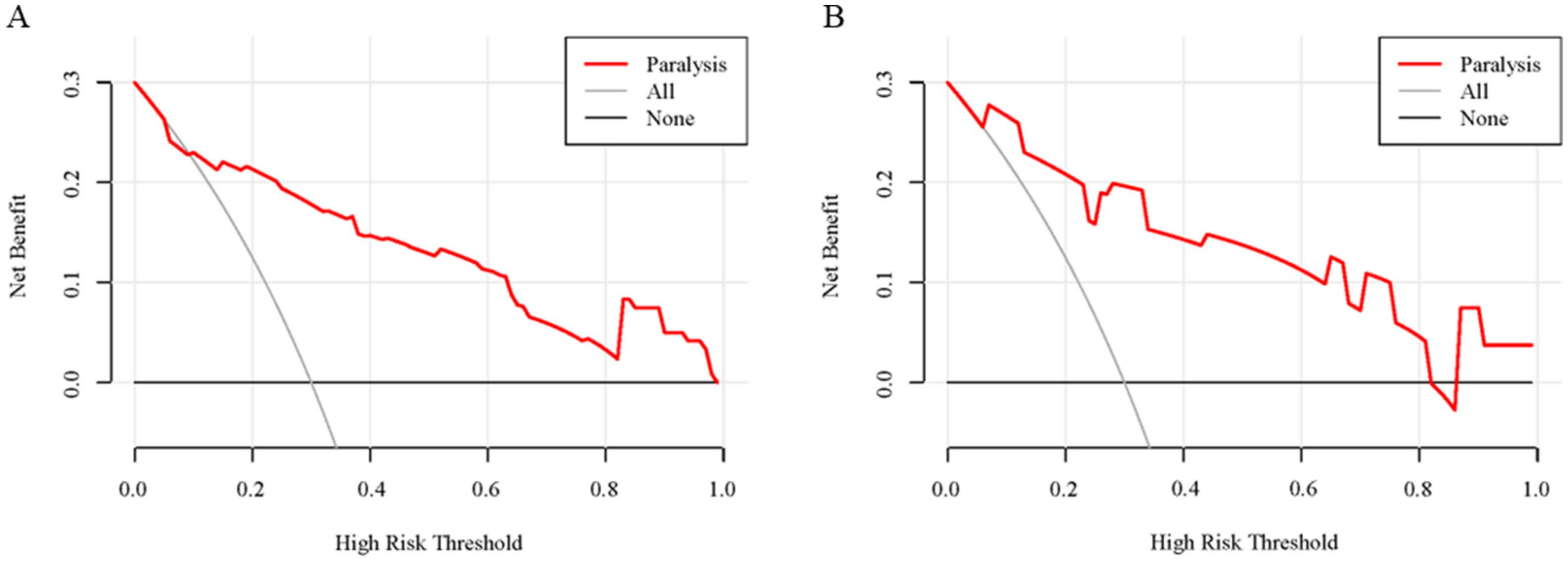
DCA of the training set **(A)** and validation set **(B)** for the nomograms. The X-axis represents the probability of paralysis occurring and the Y-axis represents the net benefit. The gray line indicates the hypothesis that all patients will be paralyzed and the black line indicates the hypothesis that all patients will be normal. The red line indicates the column line graph for paralysis prediction. The above figure predicts the rate of paralysis when the predictive model based on the nomogram is used to make clinical decisions when the threshold probabilities of the training set and validation set are >10% and 10%~80%, respectively.

DCA was used as an indicator to evaluate the validity of the clinical prognostic models, and the true-positive and false-positive differences were used to estimate the net benefit of the clinical prognostic models. Notably, DCA incorporates patient or decision-maker preferences to some extent to meet practical clinical needs. In this study, DCA showed that when the threshold probability of the training set was approximately >10% and the threshold probability of the test set was approximately 10 to 80%, the nomogram model predicted that the patient’s one-year probability of paralysis would have a greater net benefit than either “all paralyzed” or “all normal,” which somewhat strengthened the clinical utility of the prediction model. This strengthens the clinical utility of the model (Figure 6). For example, for a patient with an individual threshold probability of 60% (if the probability of paralysis is >60%, the decision maker should prevent it as early as possible). At this point, the net gain of the training and test sets is approximately 0.12. Thus, within a certain threshold probability, a model with five factors, namely, DM, damage range, spinal canal stenosis grade, postoperative complication number and surgical timing, integrated into the prediction model achieved greater clinical benefit.

## Discussion

In this study, a prognostic model based on the index of variance was compared with the specificity and sensitivity of the AISA for predicting prognostic paralysis in TCSCI patients, and the results showed that the model was more effective than the AISA in assessing prognosis. The results indicated that prognosis was associated with DM, damage range, CSCS grade, surgical timing, and postoperative complication number. In contrast, sex, age, hypertension and OPLL were not associated with prognosis. This information can inform patient treatment choices and recovery assessments.

### Sex

This study is in agreement with the majority of reports (13–18) in that it did not identify a significant effect of sex on neurological recovery. Nevertheless, estrogen has been demonstrated to exert neuroprotective effects in several studies (19–23). Many studies have indicated potential differences in neurological recovery based on sex. Sipski et al. (24) included a total of 14,433 subjects and reported that females with incomplete high quadriplegia (C1-4 level) exhibited greater FIM motor scores than males after discharge. Similarly, Sipski et al. (24) reported that females demonstrated superior neurological recovery in ASIA motor scores for complete/incomplete neurological injury from 30 days to 1 year postinjury. It has been proposed that estrogen and estrogenic compounds may also be viable treatment options (25).

### Age

Previous studies have demonstrated that younger age is a favorable prognostic factor (14, 26–28). However, there may be similar changes in ASIA motor scores between young and old individuals (13, 29–31). Harrop et al. (13) evaluated factors associated with neurological improvement in thoracic, thoracolumbar, and lumbar SCI patients and reported that age was not significantly associated with neurological improvement. Furlan et al. (31) reported no significant difference in neurological recovery between individuals aged ≥65 years and younger patients at follow-up (6 and 12 months). The present study used 65 years as a cutoff for comparison and found that age had less of an impact on patient outcomes, which may be related to paralysis grading. Wilson et al. (27) reported that older patients (≥65 years) exhibited a decrease in functionally independent measures of motor scores at follow-up, but this decrease was dependent on the AIS grade at admission. Age was found to have a greater effect in patients with AIS B and AIS C but was less important in patients with AIS A and AIS D (27). Similarly, Kramer et al. (32) reported no significant association between age and motor-level recovery in patients with tetraplegia. Thus, age may be an unfavorable factor for patients with TCSCI but has little effect on patients with complete paralysis AISA level A or AISA level D with good motor sensation, as all patients with complete nerve damage have difficulty recovering, and all patients with AISA level D return to walking. In addition to increasing the sample size, sample bias can be avoided by refining the age of the patients and the study parameters.

### Hypertension

Hemodynamic monitoring is the cornerstone of prognostic assurance (33), and prolonged nonsteady-state loading can lead to disease (34). The results of this study show that a history of chronic hypertension does not significantly adversely affect patients with TCSCI, which may be attributed to good blood pressure control after admission. In patients with chronic SCI, DM was associated with a twofold increased risk of death, whereas the relative risk of death due to hypertension was only slightly elevated (35). Animal models of severe SCI-induced hypertension indicate that this condition does not contribute to improved spinal cord blood flow and may increase the risk of hemorrhage and edema (36). Similarly, vasopressors, such as dopamine and norepinephrine, may be detrimental to spinal cord recovery and increase the risk of hemorrhage (37, 38). In their study, Kepler et al. (39) included 92 patients, 22 of whom were hypertensive. They found that a mean arterial pressure (MAP) greater than 85 mmHg was an independent risk factor for poor early neurological recovery in patients with acute SCI. This finding aligns with the results of the study by Inoue et al. (40).

### DM

In our study, DM was identified as a poor prognostic factor for TSCI patients, which is in line with previous reports by scholars that DM or hyperglycemia is a poor prognostic factor for neurological disorders in humans (41–44). A retrospective study of 219 patients revealed that individuals with DM exhibited a greater need for wheelchair assistance and a diminished capacity to ambulate within 1 year of injury (45). Furthermore, patients with motor incomplete SCI demonstrated a more suboptimal recovery of motor scores.

SCI patients who are chronically bedridden have an increased susceptibility to metabolic disorders and are at greater risk of developing DM (46). In turn, DM plays a role in accelerating functional impairment in SCI, negatively affecting motor function and histologic outcomes (47, 48), which may be attributed to neuroinflammation (47, 48), autophagy (49), oxidative stress (50), and endoplasmic reticulum stress (51, 52). DM decreases neuronal survival, promotes astrocyte proliferation, increases inflammatory cell infiltration, and inhibits neurological recovery (49).

### OPLL

OPLL reportedly increases the risk of developing TCSCI (53). Our study showed that OPLL had no significant effect on neurological recovery, but the prevalence of OPLL was 30.0% (76/253), which was much greater than the 1.9–4.3% reported in the general population in Japan and 0.1–1.7% in patients from North America and Europe (54). It has been reported that OPLL morphology is not associated with neurological outcomes in SCI patients (55, 56). Among the 129 patients studied by Okada and colleagues (57), 13 (10.1%) patients with OPLL were found to have no effect of OPLL on initial neurological status or motor recovery in TCSCI. However, it has been suggested that OPLL is an independent prognostic factor for AIS B or C patients (58), suggesting that prognosis may be affected by heterogeneity. Notably, the rate of spinal cord compression or ossification occupancy and the intramedullary signal intensity (SI) are associated with prognosis (55, 59), which reflects the mechanism by which patients with OPLL are more susceptible to TCSCI and may be the focus of future studies.

### CSCS

CSCS is a risk factor for TCSCI. The spinal cord of severe stenosis patients is more susceptible to injury than that of normal patients, which can lead to cervical instability (53, 60). A previous study reported that individuals with CSCS were 124.5 times more likely to develop SCI than were those without CSCS (61). The new grading criteria proposed by Kang and colleagues (11) in 2011 were used in this study, which were classified into four grades according to the T2-weighted sagittal images as follows: grade 0, no obvious spinal stenosis or subarachnoid compression <50%; grade 1, subarachnoid compression >50% without obvious spinal cord compression; grade 2, spinal cord compression and displacement, with no abnormal spinal cord signal; grade 3, intraspinal signal; and grade 3, intraspinal signal, without abnormal spinal cord signal. The spinal cord was not abnormal; Grade 3 indicated an abnormal signal in the spinal cord.

The sagittal diameter of the cervical canal was found to be significantly smaller in patients with tetraplegia (62). Maximum canal compromise (MCC) and maximum spinal cord compression (MSCC) have potential clinical and prognostic value. In contrast, the MCC is more accurately measured in patients with minimal spinal cord compression, whereas the MSCC is more accurately assessed in patients with severe spinal cord compression (63). A prospective study of 100 TCSCI patients by Miyanji and colleagues (64) revealed that MSCC was associated with poor neurological recovery. High cord occupancy in the spinal canal is a potential mechanism leading to the development of SCI in individuals (65), and the diameter of the canal at the level of spinal cord compression has been associated with the prognosis of patients with TCSCI (66). At ≥30% ossified material coverage, the cerebrospinal fluid space is significantly reduced, and the spinal cord lacks effective cushioning and protection (55). At >40%, severe cervical cord compression and paralysis at the time of injury are significantly increased (67).

### Damage range

The study outcomes indicated that the damage range may serve as a predictor of the neurological prognosis of patients with cervical SCI (CSCI). The larger the lesion range is, the more challenging it is for patients to achieve neurological recovery (68). Golestani et al. (7) conducted a retrospective study on 100 patients with TCSCI and reported that the length of the intramedullary lesion was a strong predictor of AIS grade conversion after decompression surgery for CSCI. The results indicated that every 1 mm and 10 mm increase in the length of the intramedullary lesion decreased the conversion rate of the AIS grade by 4 and 40%, respectively.

Furthermore, the intramedullary lesion length may be time sensitive for imaging neurological function. Matsushita et al. (69) retrospectively analyzed 102 patients with CSCI who were hospitalized within 3 days of injury. Their findings indicated that the length of the T2 high-intensity zone at 2–3 days postinjury was significantly correlated with the neurological prognosis at the time of discharge from the hospital. In contrast, the vertical diameter of the T2 high-intensity zone at 0–1 days postinjury had a weaker relationship with neurological prognosis. The correlation between the intensity zone at 2–3 days postinjury and the neurological prognosis at the time of discharge from the hospital was found to be significantly stronger than that between the vertical diameter of the T2 high-intensity zone at 0–1 days postinjury and the neurological prognosis. This is a clinically significant finding, as previous studies, including our own, did not consider the timeliness of the patient’s examination.

### Surgical timing

To date, the superiority of early surgical decompression has not been established, particularly in the context of incomplete CSCI without associated bone damage (70–72). The most intensively researched surgeries were 24 and 72 h long. As only a small number of patients underwent surgery within 24 h, the resulting sample size was insufficient to provide reliable data. We utilized 72 h postinjury as the cutoff time and demonstrated that neurological recovery was significantly greater in patients who underwent surgery within 3 days than in those who underwent surgery at a later stage.

Early surgical decompression offers a greater likelihood of neurological recovery and increased long-term survival (73–75). For patients with incomplete CSCI without severe bone damage, early surgery prevents secondary deterioration (76, 77). Furthermore, the earlier the surgery is, the better. Patients who undergo surgical decompression within 8 h have better neurological recovery than patients who undergo surgery within 8–24 h (78). Multidisciplinary guidelines led by AO Spine also recommend that early surgery be offered as an option for adult patients with acute SCI regardless of level (79). The current recommendation for the optimal timing of surgery for SCI is 24 h postinjury, which presents a significant challenge.

### Postoperative complications

Postoperative complications are a poor prognostic factor for TCSCI patients. Previous studies have reported that the most common postoperative complications after SCI are deep vein thrombosis (DVT), pulmonary complications, pressure ulcers and urinary tract infections (UTIs) (80–82). These four complications were used in this study, and the results showed that the more complications there were, the worse the patients’ neurological recovery.

### Limitations

First, it is important to acknowledge that this was a single-center retrospective study, potentially introducing a degree of bias into the outcome data. Additionally, the sample size was relatively limited. Furthermore, we did not use intraoperative ultrasound to confirm the adequacy of surgical decompression. This is something that could be improved in future prospective studies (83). Moreover, in our study, we do not perform admission activity assessments, examine injury mechanisms, or consider factors such as the presence of fractures or dislocations. This may limit the generalizability of this model. To more comprehensively elucidate the factors influencing the prognosis of TCSCI patients, it would be advantageous to perform further prospective multicenter studies with larger cohorts. Therefore, before this model can be widely adopted, it requires further validation with data from independent populations.

## Conclusion

In conclusion, this study established a scientifically robust clinical prognostic model. The sensitivity, specificity, accuracy, and clinical applicability of this model were assessed to optimize the postoperative risk of developing paralysis. The prognostic data derived from TCSCI serve as a foundation for evaluating the efficacy of novel therapies and conducting clinical trials. As our understanding of emerging prognostic factors deepens, we will be better equipped to advance targeted therapeutic interventions, thereby enhancing diagnostic and therapeutic methodologies.

## Data Availability

The raw data supporting the conclusions of this article will be made available by the authors, without undue reservation.
